# Supplementary material for: Stigma and Discrimination towards People Living with HIV in the Context of Families, Communities, and Healthcare Settings: A Qualitative Study in Indonesia
Source: Int J Environ Res Public Health. 2021 May 19;18(10):5424. doi: 10.3390/ijerph18105424 (PMC8159085; doi:10.3390/ijerph18105424)
Supplement: Supplementary file 1 [file ijerph-18-05424-s001.zip › ijerph-1218374-supplementary.pdf]

Table S1. Consolidated criteria for reporting qualitative studies (COREQ): 32-item checklist.

No	Item	Guide questions/description	
		**Domain 1: Research team and reflexivity**	
		Personal Characteristics	
1.	Interviewer/facilitator	Which author/s conducted the interview or focus group?	√
2	Credentials	What were the researcher's credentials? E.g. PhD, MD	√
3	Occupation	What was their occupation at the time of the study?	√
4	Gender	Was the researcher male or female?	√
5	Experience and training	What experience or training did the researcher have?	√
		Relationship with participants	
6	Relationship established	Was a relationship established prior to study commencement?	√
7	Participant knowledge of the interviewer	What did the participants know about the researcher? e.g. personal goals, reasons for doing the research	√
8	Interviewer characteristics	What characteristics were reported about the interviewer/facilitator? e.g. Bias, assumptions, reasons and interests in the research topic	√
		**Domain 2: study design**	
		Theoretical framework	
9	Methodological orientation and Theory	What methodological orientation was stated to underpin the study? e.g. grounded theory, discourse analysis, ethnography, phenomenology, content analysis	√
		Participant selection	
10	Sampling	How were participants selected? e.g. purposive, convenience, consecutive, snowball	√
11	Method of approach	How were participants approached? e.g. face-to-face, telephone, mail, email	√
12	Sample size	How many participants were in the study?	√
13	Non-participation	How many people refused to participate or dropped out? Reasons?	√
		Setting	
14	Setting of data collection	Where was the data collected? e.g. home, clinic, workplace	√
15	Presence of non-participants	Was anyone else present besides the participants and researchers?	√
16	Description of sample	What are the important characteristics of the sample? e.g. demographic data, date	√
		Data collection	
17	Interview guide	Were questions, prompts, guides provided by the authors? Was it pilot tested?	√
18	Repeat interviews	Were repeat interviews carried out? If yes, how many?	√
19	Audio/visual recording	Did the research use audio or visual recording to collect the data?	√
20	Field notes	Were field notes made during and/or after the interview or focus group?	√
21	Duration	What was the duration of the interviews or focus group?	√
22	Data saturation	Was data saturation discussed?	√
23	Transcripts returned	Were transcripts returned to participants for comment and/or correction?	√
		**Domain 3: analysis and findings**	
		Data analysis	
24	Number of data coders	How many data coders coded the data?	√
25	Description of the coding tree	Did authors provide a description of the coding tree?	√
26	Derivation of themes	Were themes identified in advance or derived from the data?	√
27	Software	What software, if applicable, was used to manage the data?	√
28	Participant checking	Did participants provide feedback on the findings?	√
		Reporting	

29	Quotations presented	Were participant quotations presented to illustrate the themes / findings? Was each quotation identified? e.g. participant number	√
30	Data and findings consistent	Was there consistency between the data presented and the findings?	√
31	Clarity of major themes	Were major themes clearly presented in the findings?	√
32	Clarity of minor themes	Is there a description of diverse cases or discussion of minor themes?	√
